# Resected lymph nodes and survival of patients with esophageal squamous cell carcinoma in pT2 and pT3

**DOI:** 10.1097/JS9.0000000000001023

**Published:** 2023-12-19

**Authors:** Kexun Li, Xuefeng Leng, Lin Peng

**Affiliations:** a Department of Thoracic Surgery, Sichuan Clinical Research Center for Cancer, Sichuan Cancer Hospital & Institute, Sichuan Cancer Center, University of Electronic Science and Technology of China (UESTC), Chengdu, China; b Department of Thoracic Surgery I, Key Laboratory of Lung Cancer of Yunnan Province, Yunnan Cancer Hospital, The Third Affiliated Hospital of Kunming Medical University, Cancer Center of Yunnan Province, Kunming, People’s Republic of China

*Dear Editor,*

We extend our gratitude to Dr Chen and their colleagues for devoting their time to writing an article focusing on our recently published research in the *International Journal of Surgery* ^1,2^. Our study is an observational investigation into the correlation between resected lymph nodes and the survival of patients with esophageal squamous cell carcinoma (ESCC). We appreciate their meticulous examination of our article and their insightful comments and suggestions. Chen *et al*. presented three valuable contributions and recommendations in their article.

Firstly, the significance of preoperative therapy, especially in patients with T2/T3N0-1 esophageal squamous cell carcinoma, has been acknowledged in the CROSS study. However, it is important to note that the patients included in this study underwent esophagectomy without neoadjuvant therapy from January 2010 to December 2017. Even the results of CROSS and NEOCRTEC5010 studies were used as guidelines to recommend preoperative neoadjuvant therapy for patients with locally advanced esophageal cancer^3,4^, China’s own data of esophageal squamous cell carcinoma (NEOCRTEC5010 study) was published in 2018 and the first guideline of Chinese Society of Clinical Oncology (CSCO) on diagnosis and treatment of esophageal cancer published in 2019. In the data we included from 2010 to 2017, real-world clinical practice primarily involved surgery alone for resectable tumors. However, over the past 5 years, the proportion of neoadjuvant therapy has gradually increased in our center. It is important to recognize that the exclusion of patients who received preoperative therapy may have limited the generalizability of our findings. In future studies, we will consider including patients who have undergone neoadjuvant therapy and investigate the potential impact of such treatments on the optimal number of lymph node dissection and its influence on patient overall survival (OS) outcomes.

Secondly, the challenges of accurately calculating the number of dissected lymph nodes in clinical practice are indeed significant. In another study, we addressed these complexities by describing the details of lymph node resection by node zones according to tumor location in the *Annals of Surgical Oncology* ^5^. To enhance the accuracy of N staging, we excluded patients who did not meet the minimum requirement for the number of resected lymph nodes as per the guidelines. Additionally, for enlarged confluent lymph nodes, we relied on discussions with pathologists to make estimations.

Lastly, we appreciate your observation regarding the potential bias in survival differences between the N0 and N+ groups due to the uneven distribution of T2/T3 patients in these groups. Conducting subgroup analyses based on the T stage is crucial to enhance the credibility of our study conclusions. As per your suggestion, we have included the outcomes of this analysis in Figure 1. Further subgroup analysis indicated that the patients who truly benefited were those with advanced stage (T3N+). Similarly, for node-negative patients, the addition of lymph nodes did not yield significant OS benefits.

**Figure 1:**
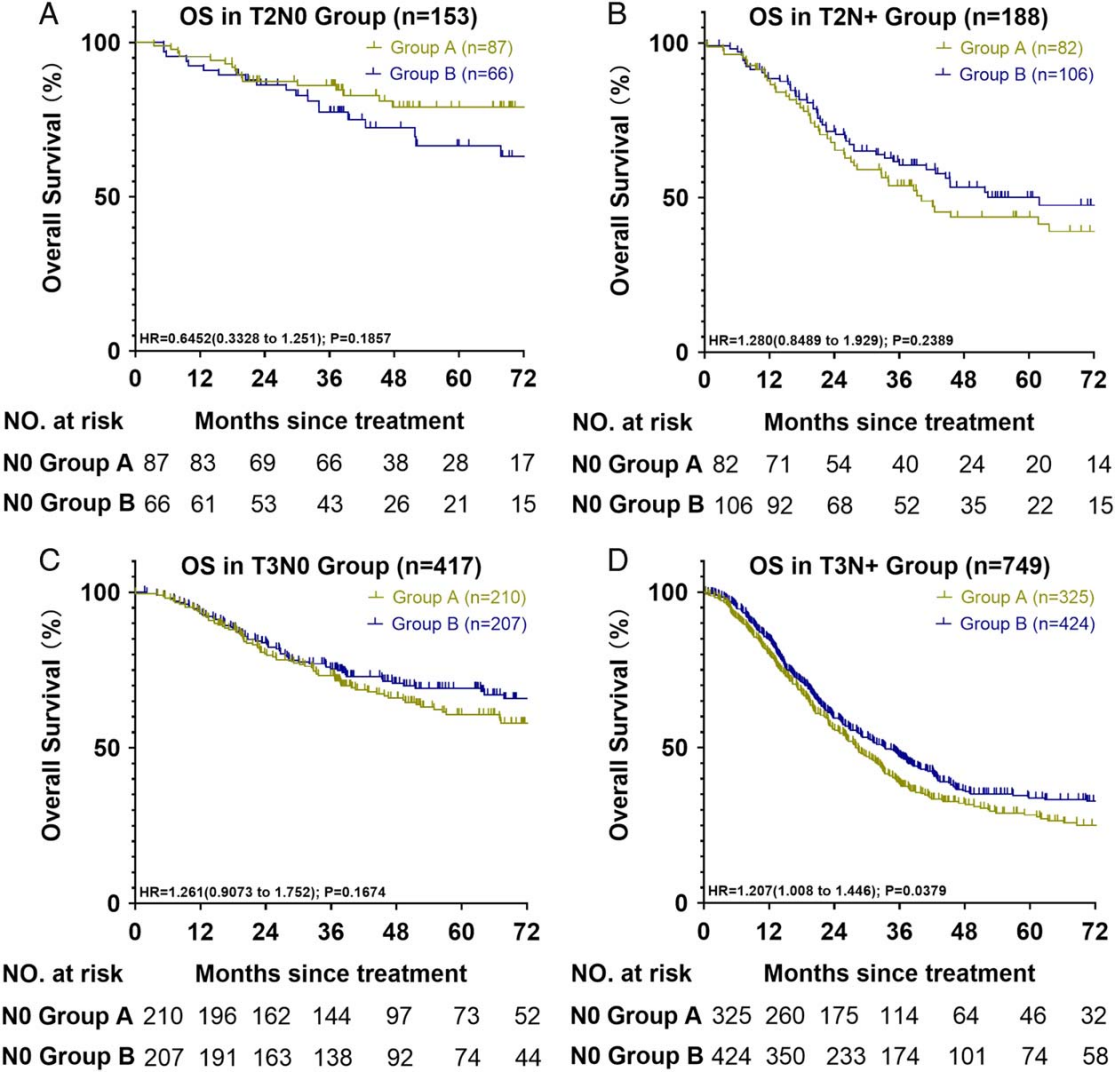
Overall survival (OS) curves of T2 and T3 participants. (A) OS curve of T2N0 patients of group A (RLNs: 15–23) and group B (RLNs: >23); (B) OS curve of T2N+ patients of group A (RLNs: 15–23) and group B (RLNs: >23); (C) OS curve of T3N0 patients of group A (RLNs: 15–23) and group B (RLNs: >23); (D) OS curve of T3N+ patients of group A (RLNs: 15–23) and group B (RLNs: >23). RNLs indicate resected lymph nodes.

We are grateful for your valuable input, and we are committed to addressing these points in our future research endeavors to enhance the robustness and clinical relevance of our findings.

Thank you once again for your thoughtful comments and for inspiring further discussion on this important topic.

## Ethical approval

All procedures performed in this study were in accordance with the Declaration of Helsinki (as revised in 2013). The study was approved by the Ethics Committee (EC) for Medical Research and New Medical Technology of Sichuan Cancer Hospital (SCCHEC-02-2022-050). Consent was waived by the Ethics Committee (EC) due to the retrospective nature of the study.

## Sources of funding

This work was supported by grants from the National Key Research and Development Program (2022YFC2403400), the Department of Science and Technology of Sichuan Province [2023YFS0044, 2020YFH0169], Sichuan Province Clinical Key Specialty Construction Project.

## Author contribution

K.L.: drafting of the article and statistical analysis; L.P. and X.L.: administrative, technical, or material support and obtained funding; K.L., X.L., and L.P.: had full access to all the data in the study and take responsibility for the integrity of the data and the accuracy of the data analysis. All authors were involved in the study concept and design, acquisition, analysis, or interpretation of data.

## Conflicts of interest disclosure

The authors have no conflicts of interest to declare.

## Research registration unique identifying number (UIN)

1. Name of the registry: ClinicalTrials.gov
2. Unique identifying number or registration ID: NCT05570487.
3. Hyperlink to your specific registration (must be publicly accessible and will be checked): https://clinicaltrials.gov/ct2/show/NCT05570487?term=NCT05570487&draw=2&rank=1.

## Guarantor

Kexun Li and Lin Peng are guarantors.

## Unblinded ethics statement

The study was approved by the Ethics Committee (EC) for Medical Research and New Medical Technology of Sichuan Cancer Hospital (SCCHEC-02-2022-050).
